# Uncertain Data Clustering-Based Distance Estimation in Wireless Sensor Networks

**DOI:** 10.3390/s140406584

**Published:** 2014-04-09

**Authors:** Qinghua Luo, Yu Peng, Xiyuan Peng, Abdulmotaleb El Saddik

**Affiliations:** 1 School of Information and Electrical Engineering, Harbin Institute of Technology at WeiHai, No.2 WenHua west road, Weihai 264209, China; 2 Guangxi Key Laboratory of Automatic Detecting Technology and Instruments, Guilin University of Electronic Technology, No.1, Jin Ji road, Guilin 541004, China; 3 Automatic Test and Control Institute, Harbin Institute of Technology, Harbin 150080, China; E-Mails: pony911@163.com (Y.P.); pxy@hit.edu.cn (X.P.); 4 Multimedia Communications Research Laboratory (MCRLab), University of Ottawa, Ottawa, ON K1N 6N5, Canada; E-Mail: elsaddik@uottawa.ca

**Keywords:** wireless sensor network, distance estimation, RSSI, uncertain data, data clustering algorithm

## Abstract

For communication distance estimations in Wireless Sensor Networks (WSNs), the RSSI (Received Signal Strength Indicator) value is usually assumed to have a linear relationship with the logarithm of the communication distance. However, this is not always true in reality because there are always uncertainties in RSSI readings due to obstacles, wireless interferences, *etc*. In this paper, we specifically propose a novel RSSI-based communication distance estimation method based on the idea of interval data clustering. We first use interval data, combined with statistical information of RSSI values, to interpret the distribution characteristics of RSSI. We then use interval data hard clustering and soft clustering to overcome different levels of RSSI uncertainties, respectively. We have used real RSSI measurements to evaluate our communication distance estimation method in three representative wireless environments. Extensive experimental results show that our communication distance estimation method can effectively achieve promising estimation accuracy with high efficiency when compared to other state-of-art approaches.

## 1. Introduction

Recently, Wireless Sensor Networks (WSNs) have attracted tremendous attention in both the research community and industry [1–3]. Precise distance estimation is needed in various WSN applications, such as velocity measurement, object identification, deployment, control, localization and tracking [4–6]. There are many available techniques to estimate distance.

Ultrasonic distance measurement methods have been proposed for accurate distance measurement [7–9], and Received Signal Strength Indicator (RSSI), Time of Arrival (TOA), Time Difference of Arrival (TDOA), and Angle of Arrival (AOA) techniques can also be used to estimate the communication distance [10]. In many WSN applications, the sensor node is sensitive to cost and power consumption, so by taking practicability, energy and cost into consideration, WSNs often adopt the low-cost Received Signal Strength Indicator (RSSI) method. In RSSI-based distance (referred to as “RSSI-D”) estimation, as is known from the ideal propagation model of radio signals, the relationship between the communication distance (*D*) and the radio signal strength is expressed by Equation (1) [11]:

(1) $$P(D)[\text{dBm}]=P(D_{0})[\text{dBm}]-10\text{nlg}(D/D_{0})-X_{r}$$

where *P*(*D*) is the signal strength indicator of an unknown node received by anchor nodes, *P*(*D*_0_) is the strength indicator of the signal sent from the reference node to the anchor node, *D*_0_ is the distance between the reference node and the anchor node, *D* is the distance to be estimated between an unknown node and anchor nodes, *n* is the channel attenuation index, which is generally equal to 2 to 4, *X_r_* is the Gaussian noise random variable.

From Equation (1), we can obtain the estimated distance *D*:

(2) $$D=D_{0}\times 10((P(D_{0})-P(D)-X_{r})/10n)$$

However, in real systems, there are uncertainties in the arriving signal strength due to the influence of environmental factors such as reflection, refraction, multi-path transmission, antenna gain, and many other obstacles [12]. Moreover, under different environments or at different communication distances, the level of uncertainty in RSSI values will also be different (if uncertainty is represented by the statistical variance, the higher the variance, the greater the uncertainty is). Generally in an open air environment the level of uncertainty in RSSI values is lower than that of an environment which has obstacles, such as walls. Therefore, the relationship between RSSI and *D* can hardly fulfill Equation (2). There is no longer a linear relationship between the RSSI value and lg(*D*) in these scenarios.

If we directly apply the above-mentioned empirical model-based linear or curve fitting method to RSSI-D estimation, the communication distance estimation relative error could be 50% or worse [13]. To solve this problem, scholars have performed many studies on the subject and have proposed various methods. Some researchers have proposed particle swarm optimization (PSO) [10], extended Kalman filter (EKF) [14,15], particle filter (PF) [16] and methodology to filter out the errors in the RSSI. However, with these filters, the system model must be accurately described and moreover, the computation complexity is high, and timing requirements in real-time processing are difficult to meet for many WSN applications. Although real RSSI values exhibit a significant level of uncertainty, their distributions still share some statistical properties in terms of uncertainties. Specifically, RSSI values with the same communication distance tend to constitute a cluster. The objective of this paper is to find a more effective way to overcome the uncertainty of RSSI values and achieve better RSSI-D estimation results.

**Figure 1.**
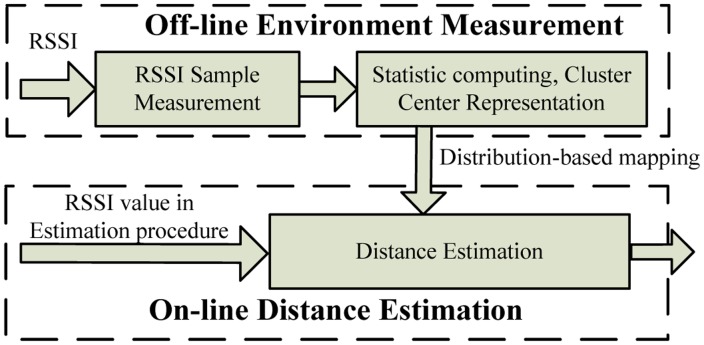
The framework of DEUDC.

To improve distance estimation accuracy, we have proposed a RSSI-D estimation method using interval data clustering, called Distance Estimation using Uncertain Data Clustering (DEUDC). As shown in Figure 1, the framework of DEUDC is comprised of an off-line environment measurement module and an on-line distance estimation module.

*Off-line environment measurement*: We first perform RSSI sample measurements at different communication points in the wireless communication environment. We then submit the RSSI data for statistical computation and model the RSSI distribution characteristic in terms of RSSI uncertainties. We can obtain an RSSI-D mapping based on this method.
*On-line distance estimation*: During the RSSI-D estimation procedure, the RSSI value is measured by a wireless sensor node (e.g., CC2530 WSN node), and we can estimate the communication distance using uncertain data clustering.

In the on-line distance estimation module, considering different levels of uncertainty in RSSI values, we adopt RSSI-D estimation methods using both hard and soft uncertain data clustering methods to improve the estimation accuracy.

The contributions of this paper are as follows:

(1) We propose DEUDC, a RSSI-based communication estimation method, which uses a mapping strategy and an uncertain data clustering method. Unlike sample-based mapping in RADAR [17] and ARIADNE [18] systems, we resort to distribution-based mapping to overcome the uncertainty in RSSI readings.
(2) To address the uncertainty in RSSI values, we adopt interval data and statistical information to represent the RSSI distribution characteristic of each distance. In comparison to sample-based mapping, by exploiting distribution-based statistics, our approach can potentially obtain greater improvement in estimation accuracy and efficiency.
(3) We propose an RSSI-D estimation method in which uncertain data soft and hard clustering algorithms are implemented in order to obtain better estimation accuracy with respect to different levels of uncertainty in RSSI.
(4) We have evaluated DEUDC using real data sets from representative wireless environment. Experimental results show that DEUDC out-performs state-of-art estimation methods.

The remainder of this paper is organized as follows: we present related work in Section 2; Section 3 introduces the uncertain data expression, including related definitions and the distance computation method used to handle interval data; Section 4 describes the RSSI-D estimation method using uncertain data clustering and its implementation; we evaluate the performance of this RSSI-D estimation method in Section 5; Section 6 concludes the paper.

## 2. Related Works

RSSI provides an inexpensive and practical way [19] of estimating communication distances during the operation of range-based localization systems or other range-based service systems used for wireless communications. Many uncertain factors exist during the measurement of RSSI [17], and the uncertainty in RSSI values leads to very low accuracy when estimating communication distances. For the RSSI-based communication distance estimation problem, many studies have been performed to improve the estimation accuracy. These studies can be divided into two categories: those dedicated to model-based methods, and those dedicated to mapping-based methods.

### 2.1. Model-Based Estimation Methods

Shang *et al.* adopted empirical models of radio propagation to estimate communication distance [20]. However, the estimation accuracy of this method is sensitive to many uncertain factors. Li *et al.* proposed a least-squares (LS) curve fitting method to reduce the influence of RSSI outliers [21]. In [22], The practice of LS-based curve fitting using a statistical means method is presented to improve the accuracy with which communication distances are estimated using RSSI, but the results are not very promising. Statistical filter methodologies, such as the extended Kalman filter [14] and particle filter [16] methodologies have been presented to improve estimation accuracy. However, with these filters, the system model needs to be accurately described; moreover, the computation complexity is high and timing requirements in real-time RSSI-D estimation are difficult to fulfill. In [23], the uncertainty in RSSI values is considered, but no further studies were performed. Kung *et al.* adopted weighted range measurements with different sensor nodes and a statistical technique to tolerate outliers [24]. CDL exploits both range-free and range-based methods to obtain better estimation quality [25].

### 2.2. Mapping-Based Estimation Methods

The RADAR system in [17] uses both empirical and mathematical models to determine RSSI-D. Results show that mapping-based empirical methods can yield better quality. The ARIADNE system, which uses cluster-based RSSI-D estimation, and does not consider the uncertainty in the RSSI values presented in [18].

### 2.3. Similar Systems

The RADAR system [17], the system most similar one to DEUDC, proposes a signal strength map (SS-MAP) and searches the location of the node. The estimation efficiency and accuracy of this system are sensitive to the number of samples. It also does not consider the uncertainty in the RSSI value.

Similarly, the ARIADNE [18] system contains two modules: a map generation module and a search module. For imprecise radio propagation map tables, the system adopts a clustering-based search algorithm to obtain a good quality of estimation. Relative to that of the RADAR system, the estimation efficiency is superior to an extent. However, when the number of samples is large, the estimation efficiency is still very low.

Unlike the aforementioned systems, in this paper we do not adopt sample-based mapping, but rather we resort to a distribution mapping strategy and use a clustering search method to estimate the communication distance. We not only consider the uncertainty in the RSSI values, but we also propose DEUDC, a communication distance estimation method that uses a clustering algorithm which can overcome the uncertainty in RSSI values in different types of environments and improve the distance estimation accuracy.

## 3. Related Definitions

We express uncertain RSSI values in terms of interval data. First, we provide some relevant definitions regarding the interval data.

**Figure 2.**
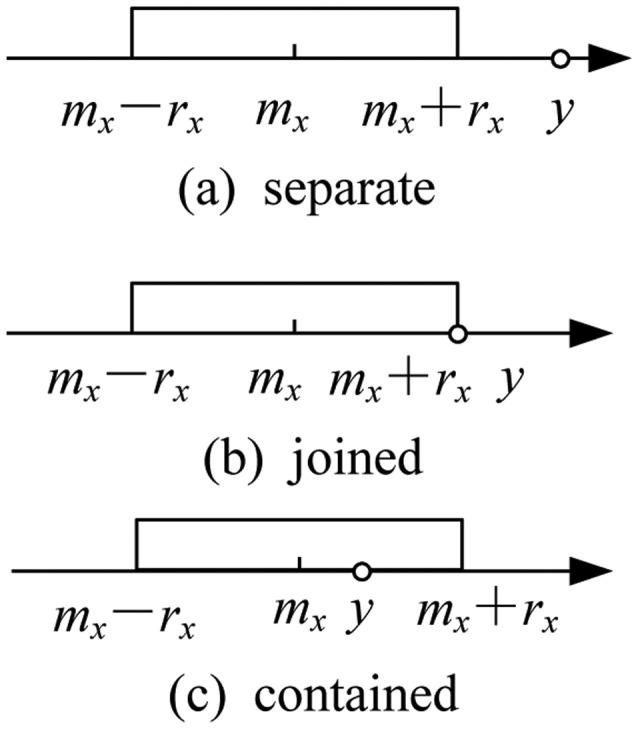
Distance relation between interval data and exact data.

(1) *Interval data* [26,27]: For given *A_L_*, *A_R_* ∈ R, and *A_R_* ≥ *A_L_*, we call the set *A* = [*A_L_*, *A_R_*] = {*u*∣*A_L_* ≤ *u* ≤ *A_R_*} interval data, where *A_L_* is the lower bound of the interval data, and *A_R_* is the upper bound. If *A_R_* = *A_L_*, which means the upper and lower bounds are equal, the interval data becomes exact data.
(2) *Midpoint and radius of interval data* [26,27]: For a given interval data *A* = [*A_L_*, *A_R_*], let *r_A_* = (*A_R_* − *A_L_*)/2; thus, we have:
  (3) $$A_{L}=m_{A}-r_{A},A_{R}=m_{A}+r_{A}$$
  We define *m_A_* and *r_A_* (*r_A_* ≥ 0) as the midpoint and radius, respectively, of interval data *A*. Therefore, we can also express the interval data as follows: [*m_A_* − *r_A_*, *m_A_* + *r_A_*].
  Because we estimate RSSI-D according to the exact RSSI values measured in the RSSI-D procedure, we propose our third definition as the distance between the interval data and the exact data.
(3) *Distance between the interval data and the exact data*: For given interval data *X* = [*m_X_* − *r_X_*, *m_X_* + *r_X_*], *Y* = *y*, where *m_X_*, *r_X_*, *y* ∈ R. The distance relationship between the two datasets is illustrated in Figure 2. When they are separate from each other, as shown in Figure 2a, the minimum distance is ∣*m_X_* − *y*∣ − *r_X_* and the maximum distance is ∣*m_X_* − *y*∣ + *r_X_*; when they are joined, as shown in Figure 2b, the minimum distance is 0, and the maximum distance is ∣*m_X_* − *y*∣ + *r_X_* = 2 *r_X_*; when the interval data contains the exact data, as shown in Figure 2c, the minimum distance is 0, and the maximum distance is ∣*m_X_* − *y*∣ + *r_X_*. Therefore, we can calculate the maximum distance *d_max_* between *X* and *Y*, the minimum distance *d_min_* and the distance *d* between the interval data and exact data as follows:
  (4) $$d_{\min}=\max(0,|m_{x}-y|-r_{x})$$
  (5) $$d_{\max}=|m_{x}-y|+r_{x}$$
  (6) $$d=[\begin{array}{cc} d_{\min} & d_{\max} \end{array}]$$

As indicated by Equation (6), the distance between the interval data and the exact data remains as interval data, which can comprehensively represent different distance values.

## 4. Algorithms for Distance Estimation Based on Uncertain Data Clustering

### 4.1. Overview of DEUDC

In this section, we first adopt the statistical information of RSSI values and interval data to represent the distribution characteristics of RSSI-D. As mentioned above, the RSSI values of the same communication distance share the same distribution characteristics and form a cluster; therefore, we can represent the distribution characteristics in the form of a cluster center. We then calculate the distance (similarity) between RSSI value and cluster centers, which will determine the RSSI value belonging to each cluster. Based on the results, taking into account the problem of different levels of uncertainty in RSSI values in different environments, we propose the RSSI-D estimation method using hard and FCM [28] soft interval data clustering algorithms.

**Figure 3.**
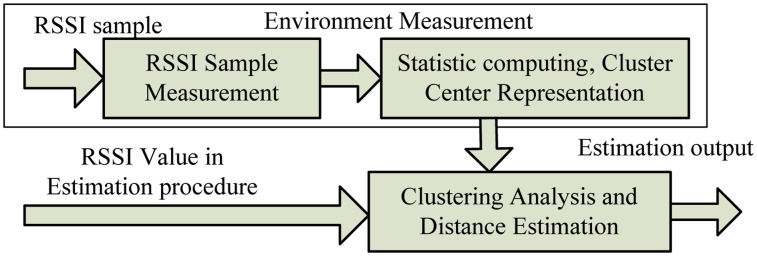
Framework of RSSI-D estimation system.

The framework of the RSSI-D estimation system is illustrated in Figure 3. The communication distance estimation system is composed of the following modules: a RSSI Sample Measure Module, a Static Computing & Cluster Center Representation Module and a Clustering Analysis & Communication Estimation Module. We first conduct environmental measurements. We then sample the RSSI values of different communication distances over certain distance intervals (e.g., 0.5 m) in the communication environment; we can then form the RSSI-D sample dataset, and submit the dataset of each communication distance to statistical computation to obtain pertinent statistical information (*i.e.*, mean and standard deviation) and express it in the form of cluster centers, which can represent the statistical RSSI-D mapping relation. During the communication distance estimation stage, we apply clustering analysis to the RSSI values according to the cluster centers and obtain the corresponding communication distance of the RSSI values.

### 4.2. RSSI Sample Measurement

In RSSI-D estimation environments (e.g., indoor corridor, hall or open air), within the communication range of the nodes, we fix the anchor node (whose position information is known) and move the unknown node relative to the anchor node by some different specific communication distances. We measure the RSSI value *Y* of different communication distances. To obtain the statistical characteristics of RSSI-D at each communication point, we measure the RSSI 150 times. Thus, we obtain the RSSI-D sample dataset. In the same manner, we perform the RSSI-D sample measurement in different types of typical communication environments, including an indoor corridor, a hall and an open air environment.

### 4.3. Statistic Computing, Cluster Center Representation

After obtaining the sample datasets, we submit the RSSI values of each communication point to statistical analysis and obtain the pertinent statistical information, namely the mean value (*μ*) and the standard deviation (*σ*). We express this statistical range as [*μ* – *k* × *σ*, *μ* + *k* × *σ*], where *k* is a coverage factor and {*k* ∈ R∣0 ≤ *k* ≤ 3}. Assume the RSSI values of every communication distance form a cluster; thus, the cluster center, or statistical region, is [*μ* – *k* × *σ*, *μ* + *k* × *σ*].

Assume the number of cluster centers for the RSSI values is *N*{*N* ∈ Z∣0 ≤ *N* ≤ 3} within the communication range, *μ_j_* is the mean value of one cluster and *σ_j_* is the standard deviation. We represent the set of cluster centers as follows: {*c_j_*}={[*μ*_1_ – *k* × *σ*_1_, *μ*_1_ + *k* × *σ*_1_], [*μ*_2_ – *k* × *σ*_2_, *μ*_2_ + *k* × *σ*_2_], …, [*μ_j_* – *k* × *σ_j_*, *μ_j_* + *k* × *σ_j_*], …,} {*N* ∈ Z∣0 ≤ *N* ≤ 3}, and the corresponding distance {*d_sj_*}.

### 4.4. Clustering Analysis and Distance Estimation

#### 4.4.1. Distance Calculation between RSSI Value and Cluster Center

To determine to which cluster the RSSI value *Y* belongs, we first define the calculation for the distance between the RSSI value *Y* and the cluster center. As stated in definition (3) in Section 3, the distance *d* = [*d_jmin_*, *d_jmax_*] between RSSI value *Y* and interval data *c_j_* = [*μ_j_* – *k* × *σ_j_*, *μ_j_* + *k* × *σ_j_*] (1 ≤ *j* ≤ *N*) is still an interval data concept. To perform clustering analysis, we introduce a correlation factor *λ* [29], where 0 ≤ *λ* ≤ 1, and use it to combine these two distance extremes to calculate the distance *D_j_*(*c_j_*, *Y*) as follows:

(7) $$D_{jY}=D_{j}(c_{j},Y)=\lambda *d_{j\min}+(1-\lambda )*d_{j\max}$$

In the equation, when *λ* is equal to 0, *D_j_*(*c_j_*, *Y*) is maximized, *i.e.*, the distance between the two sets of data is the greatest. When *λ* is equal to 1, *D_j_*(*c_j_*, *Y*) is minimized. All other values of *λ* are combinations of the two distance extremes.

#### 4.4.2. RSSI-D Estimation Method Based on Interval Data Clustering

Base on distance calculation, for an arbitrary RSSI value *Y*, we can determine to which cluster *Y* belongs using interval data clustering algorithm. We then treat the distance that corresponds to the determined cluster center as the RSSI-D estimation result. For different levels of uncertainty in RSSI value in different environments, we proposed hard-based and soft-based interval data cluster algorithm.

(1) Distance Estimation using Uncertain Data Hard Clustering (DEUDHC)
  Unlike traditional clustering analysis, the mean value *μ* and standard deviation *σ* of the cluster center are obtained through statistical calculation. Moreover, the cluster center is expressed by the interval data. For an RSSI value *Y*, in the RSSI-D estimation process, Equation (7) is used to calculate the distance between *Y* and each RSSI cluster center (*c_i_*), to determine the cluster center *c_j_* located at the shortest distance (*D_jY_*) and then use the related communication distance (*d_sj_*) of that RSSI cluster center *c_j_* as the estimated value for the communication distance (*d_c_*) of the RSSI value *Y*. We call this method Distance Estimation using Uncertain Data Hard Clustering (DEUDHC), which is based on interval data hard clustering. The main pseudo code describing how the method operates is presented in Algorithm 1.

**Figure 4.**
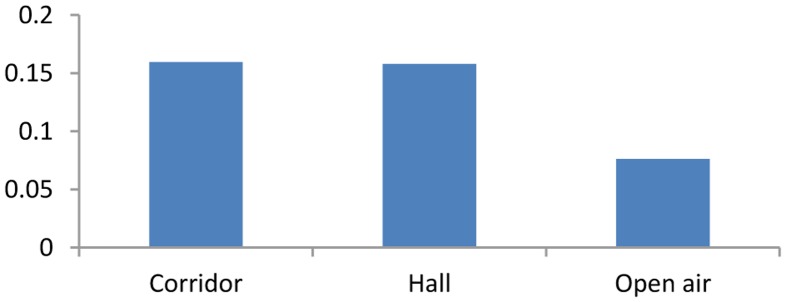
RSSI-D estimation error using DEUDHC method.

**Algorithm 1: DEUDHC ( )**
1)**Input**: *Y*, *k*, *λ*, {*c_i_*},{*d_sj_*} (0 ≤ *j* ≤ *N*) {*N* ∈ Z∣0 ≤ *N* ≤ 3}2)**Output**: *d_c_* % the communication distance estimate value of RSSI value *Y*3) while (1)4)new input *Y* % for every RSSI value5)for *j* = 1 to *N* do6)compute *D_jY_* = *D_j_*(*c_j_*, *Y*)7)end for8)find minimum *D_j_*(*c_j_*, *Y*)9)*d_c_* = *d_sj_*10) return *d_c_*11)  End
  Here, *Y* is the RSSI value used in RSSI-D estimation during the RSSI-D estimation stage, *k* is a coverage factor, *λ* is a correlation factor, {*c_j_*} {0 ≤ *j* ≤ *N*} is the center of each RSSI cluster, {*d_sj_*} {0 ≤ *j* ≤ *N*} is the communication distance related to each cluster center, and *d_c_* is the estimated value for the given RSSI value *Y*.
  When the level of uncertainty in RSSI values is very high many of the cluster centers represented by the interval data will overlap. If the DEUDHC method is adopted, the error in the distance estimation may be large. We apply the DEUDHC method for RSSI-D estimation in three typical environments, and the relative distance estimation error is shown in Figure 4, which demonstrates that the error is very large. In addition, the communication distance is discrete when using the interval number hard clustering RSSI-D estimation method because the method does not consider the RSSI value between two communication distances.
(2) Distance Estimation using Uncertain Data Soft Clustering (DEUDSC)
  To solve these problems, the distance estimation method based fuzzy clustering is introduced. We use an FCM [28] soft clustering algorithm to determine the three cluster centers that have the highest degree of membership, we denote them as *U_i_*, *U_m_*, *U_n_*. We then multiply the distances (*i.e.*, *d_si_*, *d_sm_* and *d_sn_*) related to the three cluster centers by the corresponding degrees (*i.e.*, *U_i_*, *U_m_*, *U_n_*) of membership and accumulate them (*i.e.*, *d_c_* = *U_i_* × *d_si_* + *U_m_* × *d_sm_* + *U_n_* × *d_sn_*) to obtain the estimation result *d_c_* of communication distance of RSSI value *Y*. We refer to this method as Distance Estimation using Uncertain Data Soft Clustering (DEUDSC), for which the main pseudo code is presented in Algorithm 2.

**Algorithm 2: DEUDSC ( )**
1)**Input**: *Y*, *k*, *λ*, {*c_i_*},{*d_sj_*} (0 ≤ *j* ≤ *N*) {*N* ∈ Z∣0 ≤ *N* ≤ 3}2)**Output**: *d_c_*3) while (1)4)new input *Y*5) ({*U_i_*},{*c_i_*})=FCM(*Y*, *k*, *λ*,{*c_j_*})6)find maximum *U_i_*, *U_m_*, *U_n_*7)*d_c_* = *U_i_* × *d_si_* + *U_m_* × *d_sm_* + *U_n_* × *d_sn_*8)return *d_c_*9)End


**Figure 5.**
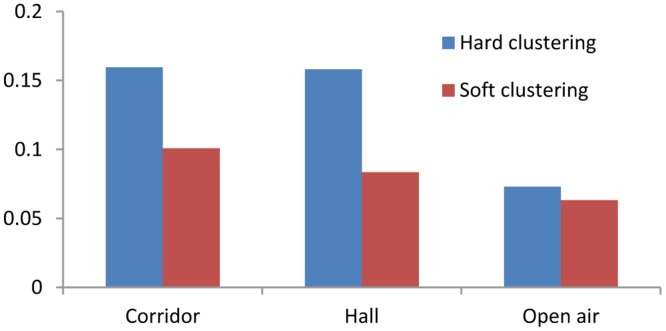
RSSI-D estimation error using interval data hard and soft clustering methods.

We apply DEUDSC to perform RSSI-D estimation in different communication environments. The relative estimation errors of the DEUDHC and DEUDSC methods are shown in Figure 5. This figure shows that the DEUDSC method can greatly improve the RSSI-D estimation accuracy relative to that of the DEUDHC method in the three typical environments under consideration. In the environments with higher levels of uncertainty in the RSSI values (*i.e.*, the corridor and the hall), the improvement in the estimation accuracy is particularly great. On the other hand, in the open air environment, which features a low level of uncertainty in the RSSI values, the improvement is very limited.

#### 4.4.3. Efficiency Improvement: Micro-Cluster Based Clustering

**Figure 6.**
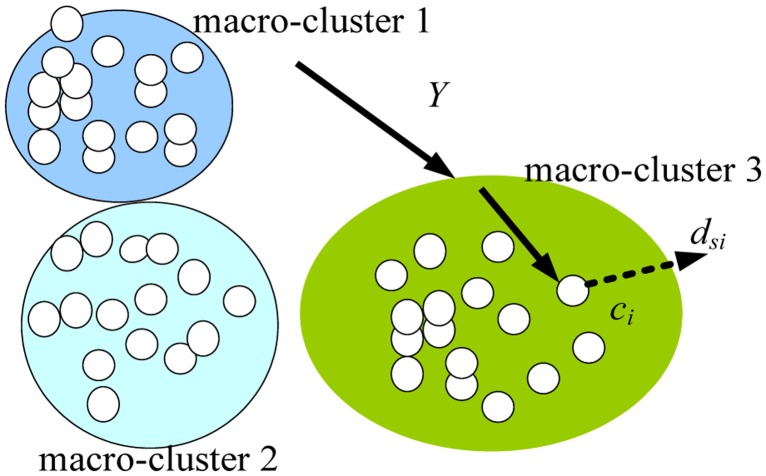
Macro-cluster and RSSI-D estimation.

To improve the efficiency of DEUDC, we apply the UK-means [30] method to perform clustering analysis on the RSSI cluster centers, and obtain macro-clusters. As shown in Figure 6, we set the number of macro-cluster centers to three, and get three macro-clusters: macro-cluster 1, macro-cluster 2 and macro-cluster 3. When we perform the RSSI-D estimation, once we obtain a RSSI value *Y*, we first determine to which macro-cluster (in this case, macro-cluster 3) the RSSI value *Y* belongs (*i.e.*, the distance between the two is minimum according to Equation (6)) [18,30]. Secondly, in macro-cluster 3, we further determine to which cluster center *c_i_* = [*μ_i_* – *k* × *σ_i_*, *μ_i_* + *k* × *σ_i_*] the RSSI value *Y* belongs according to Equation (7). Finally, we obtain the communication distance estimation result *d_si_*, which corresponds to cluster center *c_i_*. In this manner, we can improve the efficiency of RSSI-D estimation.

## 5. Experiments

In this section, we evaluate the performance of the DEUDC (including DEUDHC and DEUDSC) RSSI-D estimation method proposed in this paper. We first conduct the feasibility evaluation. In other words, we evaluate the impact of related parameters (*i.e.*, the relevant parameter *λ* and coverage factors *k*) on the performance of the RSSI-D estimation method in different environments to obtain the appropriate setting of these parameters. Second, we evaluate the performance of the DEUDC RSSI-D method in three typical environments, and compare with other RSSI-D estimation methods. Finally, we discuss the experimental results and draw general conclusions.

### 5.1. Experiment Setup

#### 5.1.1. Experiment Setting and Experimental Data

**Figure 7.**
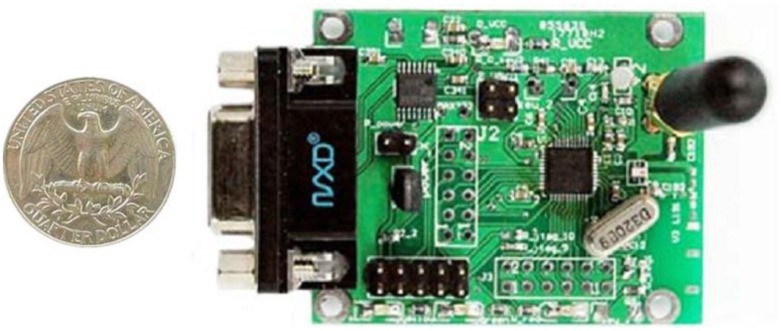
The node used in experiment.

The experimental conditions and parameter settings are shown in Table 1. We design CC2530 WSN nodes based on the TI (Texas Instruments Corporation, Dallas, TX, USA) System on Chip (SOC) framework, shown in Figure 7, and use them for our experiments.

**Figure 8.**
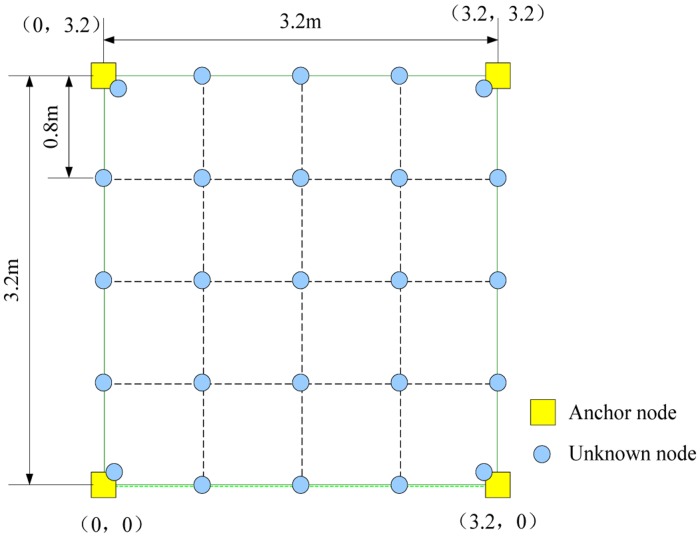
The deployment of the distance estimation field.

We deploy a real distance estimation system in a 3.2 m × 3.2 m field with sensor nodes, as shown in Figure 8. We fix the four anchor nodes and move the location of unknown node at intervals of 0.8 m in two directions (when it overlaps with an anchor node, we move the unknown node 0.1 m from the anchor node). We deploy the system in different environment, e.g., in a corridor, a hall and an open air environment.

The configuration of evaluation platform (PC) is as follows. CPU: Intel i7 720QM@1.6 Ghz, main memory: 4 GByte, Operating system: Window XP Professional SP3. Evaluation environment: Matlab 2009b.

After deploying the distance estimation system, we perform the following sampling procedure and get experimental data:

Step 1: At each location point, after receiving RSSI-D estimation request from the sink node (connected to a PC and managing the WSN network), the unknown node sends an RSSI request signal to the anchor nodes.
Step 2: The anchor nodes measure the RSSI value of the request signal and send it to the unknown node.
Step 3: After receiving these RSSI values from the four anchor nodes, the unknown node sends them to the sink node.

At each of these 25 points, we repeat the sample procedure 150 times to obtain the RSSI values of the link between the unknown node and the four anchor nodes. We then perform statistical computation and thus obtain 25 RSSI-D mapping models. After modeling, we sample the RSSI values 50 times at each of the 25 RSSI-D estimation points and perform RSSI-D estimation using the RSSI-D mapping models.

#### 5.1.2. Evaluation Metrics

We evaluate the estimation accuracy and estimation efficiency of the RSSI-D method in terms of the following metrics.

(1) Estimation accuracy metric
  For estimation accuracy, in this experiment, we adopt the following metric: the RSSI-D estimation absolute error (*AE*) as indicated in Equation (8):
  (8) $$AE=|d_{t}-d|$$
  where *d_t_* is the RSSI-D estimation value (*i.e.*, distance estimation value) between an unknown node and anchor nodes, *d* is the real distance value between an unknown node and anchor nodes, *AE* is the RSSI-D absolute error.
  The lower the values of these parameters, the more accurate the results become. We perform the following evaluation based on the metric.
(2) Estimation efficiency metric

For estimation efficiency, we adopt the following metrics: model time *T_m_* (modeling time) and *T_e_* (estimating time). Low values of these parameters means that the estimation efficiency is high.

### 5.2. Feasibility Evaluation

In this section, we evaluate the impact of important parameters (*i.e.*, correlation factor *λ* and coverage factor *k*) and the appropriate setting of these parameters.

#### 5.2.1. Impact of Correlation Factor on the RSSI-D Estimation Method

**Figure 9.**
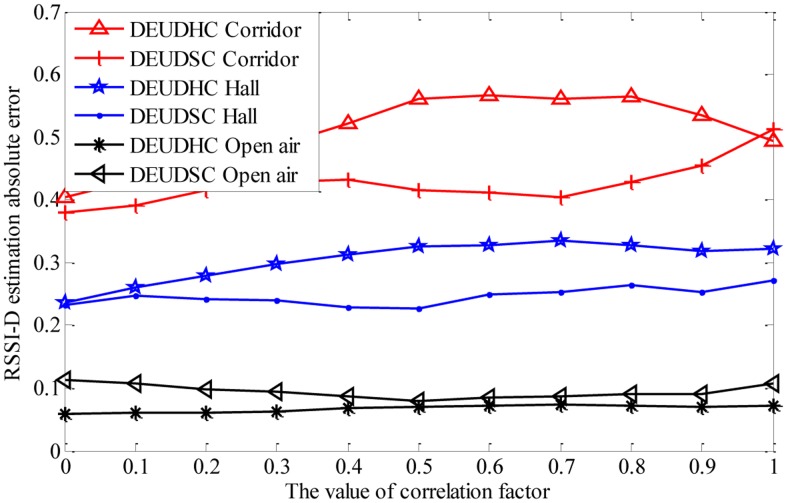
Changes in distance estimation errors with correlation factor in three environments.

(1) Impact of correlation factor
  The correlation factor *λ* determines the combination of the maximum and minimum distance between RSSI value *Y* and the cluster center during the distance calculation in the clustering process (shown in Equation (7)), and 0 ≤ *λ* ≤ 1. We use different values of the correlation factor *λ* in the experiment to evaluate the factor's impact on the performance of the RSSI-D estimation method. The conditions are listed in Table 2. We fix the anchor node and move the unknown node shown in Figure 8. We set the value of the coverage factor *k* to 1 and apply the DEUDHC method to perform RSSI-D estimation. Figure 9 shows the mean *AE* (absolute error of all communication distances) of the RSSI-D estimation.
  From Figure 9, we could see that the correlation factor does not have a clear impact on the RSSI-D estimation accuracy. For different values of the correlation factor *λ*, the estimation error does not vary appreciably. Therefore, we can set *λ* to a random value. To obtain better RSSI-D estimation results, in this experiment, we should set the values of the correlation parameter *λ* to be 0 to 0.1, 0 to 0.1 and 0.5 to 0.6 when in a corridor, a hall and an open air environment, respectively.
(2) Discussion on setting of correlation factor

Based on the experimental results and analysis described above, the impact of the correlation factor on RSSI-D varies based on the different environments. Thus, when we apply the DEUDC RSSI-D estimation method, we should analyze the correlation factor setting procedure through experiments.

#### 5.2.2. Impact of Coverage Factor on the RSSI-D Estimation Method

Coverage factor *k* determines the range of interval data. According to the error theory [31], when the value of *k* is greater than three, we treat the data as outliers. By considering the cluster centers' representative form as interval data, we can see that when *k* is too large, the range of cluster centers will be too wide, which leads to serious overlap between cluster centers and, therefore, a larger RSSI-D estimation error. Therefore, *k* should take on a smaller value.

**Figure 10.**
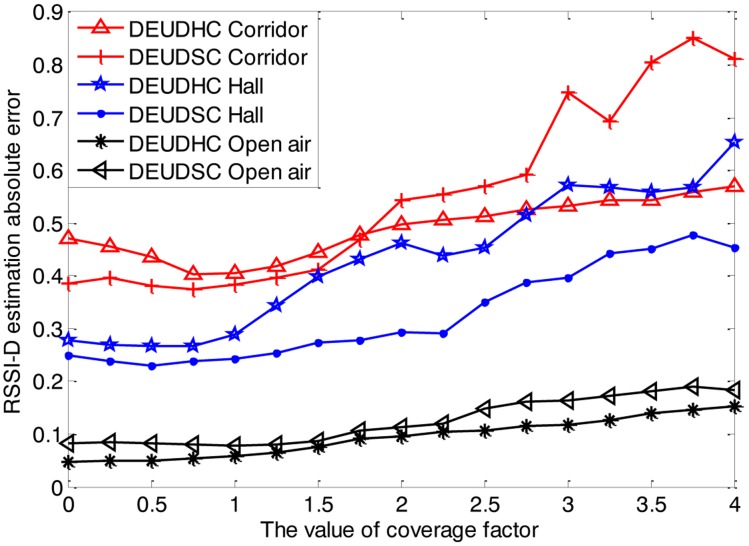
Changes in distance estimation error with coverage factor in three environments.

(1) Impact of *k*
  To evaluate how the coverage factor *k* affects the performance of DEUDC method, we adopt different coverage factor values and apply the DEUDHC and DEUDSC methods for RSSI-D estimation in the three environments mentioned above. According to the impact analysis of the correlation parameter *λ*, the correlation factor in the DEUDHC and DEUDSC estimation methods *λ* takes on values of 0.1 and 0.1, 0.1 and 0.1 and 0.5 and 0,5, respectively, in the corridor. The RSSI-D estimation error of each node is shown in Figure 10.
  Figure 10 shows that the RSSI-D estimation error increases with *k*. Therefore, *k* should take on values of 0.75 to 1.25 when in a corridor, 0.25 to 0.75 when in a hall and 0 when in open air.
(2) Analysis of relation between the RSSI standard deviation and the value of *k*
  We calculate the standard deviations of the RSSI values of each communication distance point in the three environments. We also evaluate the effect of *k* in three typical environments and determine the appropriate value of *k*, as shown in Table 3.
  The standard deviation represents the level of fluctuation in measurement data [32]. In this paper, we use the standard deviation to index the uncertainty level in RSSI values. Table 3 shows that the level of uncertainty in the RSSI values is high in the corridor, while that of uncertainty is low in open air. This is because radio reflection, refraction, diffraction and multi-path propagation occur in the hall, while there exists few of these uncertain cases.
(3) Discussion on setting of coverage factor *k*

The results of the value-setting experiments performed for the coverage factor *k* in different environments demonstrate that the appropriate values of *k* are closely related to the level of uncertainty in RSSI values. Generally, when the standard deviation of RSSI value is about 2, *k* takes on a value of 1, and *k* take on a value of 0.5 and 0, when the standard deviation of RSSI value is above 1 and below 1, respectively. We should determine the most suitable value of k through experimental analysis when we apply the RSSI-D estimation method.

### 5.3. Performance Evaluation

In this section we evaluate the performance of the RSSI-D DEUDC (including DEUDHC and DEUDSC) method. We apply the following RSSI-D estimation methods to estimate the distances in the three typical environments: Least Square Linear Fitting (LSLF) [31,33,34], Step Regression Linear Fitting (SRLF) [34], Back Propagation (BP) [35], Least Square-Support Vector Machine (LS-SVM) [34] and DEUDC (proposed in this paper, including DEUDHC and DEUDSC).

In LSLF method, the mean of RSSI sample value with every distance is used to fit a linear curve (as shown in Equation (1)) with least-square rule. And the curve is regarded as the radio propagation model. Based on the model, the distance estimation result can be obtained, given a RSSI value in estimation procedure. In SRLF method, the mean of RSSI sample value is used to model the radio propagation using step linear regression method. BP is a kind of Artificial Neural Network (ANN), which is widely used in data pattern recognition. In the Back Propagation (BP) method, the RSSI-D mapping model is obtained by training the neural network using RSSI-D sample data sets, and the parameter setting is shown in Table 4, then we can get the distance estimation result by simulate the model using RSSI data. In LS-SVM method, RSSI-D sample dataset is mapped into feature space by kernel function, and model is trained in the feature space. Based the model, we can get distance estimation result, and the parameter setting is shown in Table 5. Based on the results obtained from the analysis of the correlation factor and coverage factor in Section 5.2, we set the parameters as shown in Table 6.

**Figure 11.**
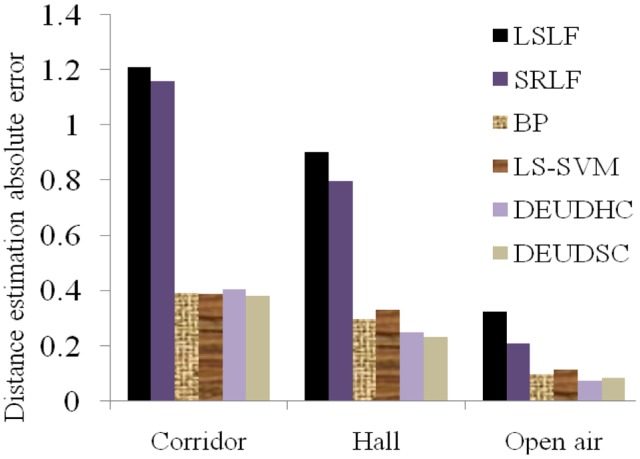
RSSI-D estimation absolute error of different methods in three typical environments.

(1) Accuracy Analysis of RSSI-D estimation
  After RSSI-D estimation, we calculate the mean of the RSSI-D estimation absolute error (AE) for each method in the three typical environments. Table 7 and Figure 11 show the RSSI-D estimation error of the different methods. We perform the following analysis:
  Table 7 and Figure 11 indicate that the DEUDC (including DEUDHC and DEUDSC) method proposed in this paper achieves higher estimation accuracy than the other methods in the three typical environments on average. Specifically, compared to LSLF, SRLF, BP and LS-SVM, the DEUDC could improve the RSSI-D estimation accuracy by 11.31% to 72.15%. Therefore, the DEUDC could overcome the uncertainty problem associated with RSSI value and reduce the RSSI-D estimation error to achieve higher estimation accuracy.
  Table 7 and Figure 11 demonstrate that the environments have a great impact on the RSSI-D estimation accuracy. For example, the corridor and the hall may feature reflection, inflection, multipath propagation and other uncertain factors which result in more complex communication environments and lead to lower RSSI-D estimation accuracy. On the other hand, in the open air environment, there exist fewer uncertain communication factors; thus, the RSSI-D estimation accuracy is higher.
(2) Efficiency Analysis of RSSI-D estimation
  Based RSSI-D estimation, we also evaluate the estimation efficiency in terms of modeling time, estimation time and total time, and Table 8 shows the estimation efficiency of different methods.
  Table 8 demonstrates that the DEUDC method proposed in this paper achieves higher estimation efficiency than most of other methods. More specifically, compared to SRLF, BP and LS-SVM method, DEUDHC can improve the estimation efficiency on the scale of 98.59%, 99.99% and 99.97% respectively. And DEUDSC can improve the estimation efficiency on the scale of 85.80%, 99.87% and 99.69% respectively.
  LSLF method uses RSSI-D sample data to fit certain linear model, so the efficiency is very high. In SRLF method, step regression strategy is used to fit linear model, so estimation efficiency is low. The estimation efficiency of BP and LS-SVM is low, that is because the modeling and computation is complex. In DEUDC method, the modeling and estimation is simple, so its efficiency is higher. So DEUDC is more suitable for applying in WSN.
(3) Discussion on innovation
  From Figure 11, we can see that, compared with BP and LS-SVM methods, the estimation accuracy improvement of DEUDC is limited. However, from estimation efficiency point of view, the improvement is very obviously. Considering the estimation accuracy and efficiency, the performance evaluation results indicate that, compared to the LSLF [31], SRLF [34], BP [35] and LS-SVM [36] methods, the DEUDC (including DEUDHC and DEUDSC) method exhibits higher estimation performance in the three typical environments. This result is observed, because the curve fitting based methods (e.g., LSLF and SRLF) assume that the RSSI values are related to the communication distance, though the relation does not exist, which leads to lower RSSI-D estimation accuracy.
  On the other hand, the RSSI-D estimation method DEUDC based on interval data clustering considers the distribution characteristics of RSSI values in real communication environments and builds a mapping relation between RSSI and distance (D), which leads to a higher performance.
  This method is not only suitable for RSSI-D estimation in wireless sensor networks, but can also be applied in other radio transmission systems.
(4) Discussion on DEUDC method and application environment

The experimental results demonstrate that in the corridor and hall, where the level of uncertainty of the RSSI values is higher, the RSSI-D estimation error of DEUDSC is lower than that of DEUDHC. On the other hand, in the open air environment, where the level of uncertainty in RSSI values is lower, the RSSI-D estimation error of DEUDSC is lower than that of DEUDHC. Therefore, we should select the RSSI-D estimation method that best suits a given communication environment.

### 5.4. Discussion on Generality of DEUDC RSSI-D Estimation

#### 5.4.1. Generality of DEUDC Distance Estimation

It should be noted that, the off-line environment measurement module in DEUDC method is not necessary, *i.e.*, the DEUDC method can be applied beyond already known and measurement environments, if application requirements are not sensitive to estimation accuracy, we can estimate the RSSI-D distance with the help of empirical radio propagation model.

#### 5.4.2. Implementation of Distributed DEUDC RSSI-D Estimation

In this paper, we focus on the evaluation and analysis of distance estimation method (*i.e.*, DEUDC) based on RSSI with different levels of uncertainty. So we resort to central processing strategy to do distance estimation. And more, the distance estimation method can be implemented distributed. In the distributed system, the RSSI-D estimation can be performed on each unknown node in WSN.

## 6. Conclusions

Targeted for communication distance estimation in real WSN applications, we have proposed a RSSI-D estimation method, DEUDC, which utilizes uncertain data clustering algorithms. The key idea is the leverage of interval data combined with the statistical distribution of RSSI values, followed by distance estimation using interval data clustering algorithms. Extensive experimental results show that the DEUDC RSSI-D estimation method can largely overcome the uncertainty of RSSI values in a real system while achieving promising RSSI-D estimation accuracy, whereas the improvement is more evident in environments where RSSI readings have larger uncertainties. The DEUDC method can provide precise distance estimation for not only localization but also object identification, deploy, item tracking and many others.

For the sake of good estimation accuracy, the RSSI-based distance estimation method requires that wireless measurements should be performed in advance, which essentially will become a bottleneck if wireless measurements are not feasible. For future works, we may explore the adaptive WSN RSSI-D estimation methods, in which a maximum likelihood or least-square method can be used to update model parameters iteratively in a real-time manner. We note there are fundamental challenges for adaptive estimation methods too, e.g., computation costs, energy consumption, *etc.*, which will be left as our future work.

## Figures and Tables

**Table 1.** Experiment conditions and parameters.

**Parameter**	**Environment**		

	**Corridor**	**Hall**	**Open Air**
Node	CC2530	CC2530	CC2530
Temperature	23.0 °C	22.5 °C	19.0 °C
Height of node	0.1 m	0.1 m	0.1 m
RSSI-D estimation field	3.2 m × 3.2 m	3.2 m × 3.2 m	3.2 m × 3.2 m
RSSI-D estimation points	25	25	25

**Table 2.** The experimental conditions.

**Parameter**	**Environment**		

	**Corridor**	**Hall**	**Open Air**
Deploy	25 RSSI-D estimation points		
The value of *k*	1	1	1

**Table 3.** The experimental parameters and results.

**Parameter**	**Environment**		

	**Corridor**	**Hall**	**Open Air**
Standard deviations of RSSI	2.31	1.31	0.49
The values of *k*	1	0.5	0

**Table 4.** The parameters setting of BP method.

**Parameter**	**Value**
neurons of hidden layer	10
epoch time	0.25
learning function	LEARNGN
goal	10^−3^

**Table 5.** The parameters setting of LS-SVM method.

**Parameter**	**Value**
gam	250
sig2	300
kernel	RBF

**Table 6.** The experimental parameters and results.

**Parameter**	**Environment**					

	**Corridor**		**Hall**		**Open Air**	

	**Hard**	**Soft**	**Hard**	**Soft**	**Hard**	**Soft**
The value of *k*	1.0	1.0	0.5	1.5	0	0
The value of *λ*	0.1	0.1	0.1	0.1	0.5	0.5

**Table 7.** The experimental results of different RSSI-D methods.

**RSSI-D Estimation Methods**	**Environment**		

	**Corridor**	**Hall**	**Open Air**
LSLF	1.21 m	9.02E-1 m	3.23E-1 m
SRLF	1.16 m	7.99E-1 m	2.08E-1 m
BP	3.92E-1 m	2.95E-1 m	9.63E-2 m
LS-SVM	3.87E-1 m	3.32E-1 m	1.15E-1 m
(Proposed) DEUDHC	4.06E-1 m	2.49E-1 m	7.54E-2 m
(Proposed) DEUDSC	3.82E-1 m	2.35E-1 m	8.41E-2 m

**Table 8.** Efficiency analysis of different RSSI-D methods.

**Method**	**Time (s)**		

	**Modeling**	**Estimation**	**Total**
LSLF	0.0060	0.0003	0.0063
SRLF	0.2765	0.0003	0.2768
BP	30.0560	0.1118	30.1678
LS-SVM	11.9440	0.7122	12.6563
(Proposed) DEUDHC	0.0026	0.0013	0.0039
(Proposed) DEUDSC	0.0026	0.0367	0.0393
